# Rates of Overall Survival and Intracranial Control in the Magnetic Resonance Imaging Era for Patients With Limited-Stage Small Cell Lung Cancer With and Without Prophylactic Cranial Irradiation

**DOI:** 10.1001/jamanetworkopen.2020.1929

**Published:** 2020-04-01

**Authors:** Todd A. Pezzi, Penny Fang, Olsi Gjyshi, Lei Feng, Suyu Liu, Ritsuko Komaki, Steven H. Lin

**Affiliations:** 1 Division of Radiation Oncology, The University of Texas MD Anderson Cancer Center, Houston; 2 Division of Biostatistics, The University of Texas MD Anderson Cancer Center, Houston

## Abstract

**Importance:**

Historical data suggest that there is an overall survival benefit associated with prophylactic cranial irradiation (PCI) in patients with small cell lung cancer (SCLC). However, as the fidelity of magnetic resonance imaging (MRI) of the brain continues to improve, this idea is now being questioned, with recent research showing no survival benefit associated with PCI in extensive-stage SCLC; however, the role for PCI is not clear in patients with limited-stage SCLC (LS-SCLC).

**Objective:**

To report the overall survival and rates of intracranial control for patients with LS-SCLC, all staged with MRI, who either did or did not undergo PCI.

**Design, Setting, and Participants:**

This cohort study included 297 patients with LS-SCLC at a large US academic cancer center. Patients were treated with thoracic radiation; 205 also underwent PCI and 92 did not. All patients underwent at least baseline MRI, with restaging brain MRI and/or computed tomography; they did not have disease progression after thoracic radiation treatment. A propensity score–matching analysis was undertaken in an attempt to adjust for potential bias. Of the 297 patients who met the inclusion criteria, the propensity score was calculated for 295 patients, using patient, tumor, and treatment characteristics. Data were analyzed in October 2019.

**Intervention:**

Prophylactic cranial irradiation in patients with LS-SCLC.

**Main Outcomes and Measures:**

The rate of overall survival and intracranial control.

**Results:**

Of the 297 patients, 162 (54.5%) were men. The median age was 62.2 years (range, 27.0-85.0 years) for patients who underwent PCI and 68.6 years (range, 40.0-86.0 years) for those who did not undergo PCI. The 3-year cumulative incidence rate of brain metastases was higher in the no-PCI group vs the PCI group, when counting death as a competing risk, but the difference was not statistically significant (20.40% [95% CI, 12.45%-29.67%] vs 11.20% [95% CI, 5.40%-19.20%]; *P* = .10). The use of PCI was not associated with a difference in overall survival between the patient groups (hazard ratio, 0.844; 95% CI, 0.604-1.180; *P* = .32).

**Conclusions and Relevance:**

These findings suggest that patients with LS-SCLC staged with MRI who undergo PCI after thoracic radiation treatment were not associated with a decreased risk of developing new brain metastases compared with patients who do not undergo PCI. The use of PCI was not associated with an overall survival benefit for such patients.

## Key Points

**Question:**

After staging with magnetic resonance imaging, is there a benefit associated with prophylactic cranial irradiation (PCI) for patients with limited-stage small cell lung cancer?

**Findings:**

In this single-institution cohort study, a propensity-matched analysis of 297 patients was conducted. The 3-year cumulative incidence rate of brain metastases was higher in the group that did not undergo PCI vs the group that did undergo PCI, but the difference was not statistically significant; PCI was not associated with an overall survival benefit.

**Meaning:**

Given the neurocognitive toxic effects associated with whole-brain radiation therapy, these data suggest that the benefits of PCI for unselected patients with limited-stage small cell lung cancer are limited.

## Teaser

This cohort study analyzes the overall survival and risk of brain metastasis associated with prophylactic cranial irradiation among patients with limited-stage small cell lung cancer.

## Introduction

Recently, the benefit of prophylactic cranial irradiation (PCI) in the setting of extensive-stage small cell lung cancer (SCLC) was called into question with the publication of a Japanese randomized trial^1^ demonstrating worse survival with PCI, in an era of magnetic resonance imaging (MRI) staging and surveillance. In contrast, in a randomized European Organisation for Research and Treatment of Cancer trial^2^ conducted during the pre-MRI era, PCI was found to provide an overall survival (OS) benefit. A recent meta-analysis^3^ of 7 similar randomized trials, which included more than 2000 patients, found substantial heterogeneity in their OS analysis, which the authors thought was associated primarily with the heterogeneity in imaging protocols among the various trials. Analogously, we hypothesize that the demonstrable utility of PCI for limited-stage SCLC (LS-SCLC) may also be diminished in patients treated in an era of contemporary imaging and modern, aggressive salvage techniques.

Currently, PCI is a category 1 recommendation after at least a partial response to thoracic radiation, according to the National Comprehensive Cancer Network and the American Society for Radiation Oncology guidelines.^4^ These recommendations are based primarily on a large meta-analysis^5^ of LS-SCLC trials showing a 5.4% OS benefit associated with PCI. However, most of the data on this topic, including that meta-analysis, were published before the routine use of MRI for staging, which has superior sensitivity in detecting brain metastasis (BM) compared with computed tomography.^6^ In this analysis, we compared the rates of intracranial control and OS data in patients with LS-SCLC, all of whom underwent staging with MRI, who were treated with or without PCI.

## Methods

This cohort study was approved by the institutional review board of the University of Texas MD Anderson Cancer Center. The need for informed consent was waived because the data were deidentified. This study follows the Strengthening the Reporting of Observational Studies in Epidemiology (STROBE) reporting guideline.

We performed a retrospective review of patients with LS-SCLC treated from 1992 to 2012. All patients underwent at least baseline brain MRI and restaging brain MRI and/or head computed tomography, and none of them had disease progression after thoracic radiation treatment; 205 patients underwent PCI and 92 did not. Of the 297 patients who met the inclusion criteria, we calculated the propensity score for 295 patients using age, sex, Eastern Cooperative Oncology Group performance status, tumor size, and radiation dose to the chest with a greedy 5:1 digit matching algorithm.^7^ The postmatch balance was assessed using standardized differences.

### Statistical Analysis

Two-sided Fisher exact, χ^2^, and Wilcoxon rank sum tests were used to compare baseline characteristics. *P* < .05 was considered statistically significant. Kaplan-Meier and Cox proportional hazards models were used to evaluate the association between PCI and OS. The Schoenfeld residual was used to check the proportional hazards assumption. To quantify the risk of developing distant BM, we also used the cumulative incidence function and counted death as a competing risk. To evaluate the association between PCI and the cumulative incidence of BM with the adjustment from other prognostic factors (mentioned later in the Results section), we used the multivariable proportional hazards models of Fine and Gray.^8^ SAS statistical software version 9.4 (SAS Institute), S-Plus statistical software version 8.2 (TIBCO Software Inc), and R statistical software version 3.4.3 (R Project for Statistical Computing) were used. Data were analyzed in October 2019.

## Results

Overall, 162 of 297 patients (54.5%) were men, and 261 of 297 patients (87.8%) had Eastern Cooperative Oncology Group performance status of 0 or 1.^9^ Baseline characteristics are outlined in Table 1. The median age was 62.2 years (range, 27.0-85.0 years) for patients who underwent PCI and 68.6 years (range, 40.0-86.0 years) for those who did not undergo PCI. The median thoracic radiation treatment dose in both groups was 45.0 Gy (range, 45.0-70.0 Gy for the PCI group and 32.4-70.0 Gy for the no-PCI group). Nearly all patients received 25 to 30 Gy in 10 to 15 total fractions for PCI. Sixty-four patients (91.4%) in the no PCI group vs 93 (94.9%) in the PCI group had a complete or partial response to radiation therapy (missing data were excluded for the calculation of these proportions). The mean (SD) primary tumor size was 4.15 (2.50) cm for the no-PCI group and 4.35 (2.58) cm for the PCI group. Sixty-seven of 81 patients (82.7%) in the PCI group and 34 of 42 patients (81.0%) in the no-PCI group received cisplatin and etoposide as opposed to an alternative systemic therapy regimen. The median estimated duration of follow-up was similar for the 2 groups (83.64 months in the PCI group and 83.97 months in the no-PCI group; overall range, 2.5-235.0 months).

**Table 1.** Descriptive Characteristics Before and After Propensity Score Matching

Characteristic	Participants, No. (%)^a^						
	Before propensity score matching			After propensity score matching			
	No PCI (n = 92)	PCI (n = 205)	*P* value	No PCI (n = 84)	PCI (n = 84)	*P* value	Mean standardized difference, %
Age, median (range), y	68.6 (40.0-86.0)	62.2 (27.0-85.0)	.001	67.5 (40.0-86.0)	65.0 (44.0-85.0)	.67	1.03
Sex							
Female	40 (43.5)	95 (46.3)	.65	37 (44.0)	35 (41.7)	.76	4.78
Male	52 (56.5)	110 (53.7)		47 (56.0)	49 (58.3)		
Eastern Cooperative Oncology Group performance status							
0	20 (21.7)	52 (25.4)	.02	19 (22.6)	19 (22.6)	>.99	1.78
1	53 (57.6)	136 (66.3)		53 (63.1)	52 (61.9)		
2	15 (16.3)	15 (7.3)		10 (11.9)	11 (13.1)		
3	4 (4.3)	2 (1.0)		2 (2.4)	2 (2.4)		
Chemotherapy regimen							
Cisplatin or etoposide	34 (81.0)	67 (82.7)	.81	32 (82.1)	29 (82.9)	.93	NA
Other regimen	8 (19.0)	14 (17.3)		7 (17.9)	6 (17.1)		
Smoker							
No	8 (10.8)	36 (22.2)	.12	8 (11.8)	16 (25.8)	.10	NA
Yes	66 (89.2)	126 (77.8)		60 (88.2)	46 (74.2)		
Largest dimension of primary tumor, cm							
Mean (SD)	4.15 (2.50)	4.35 (2.58)	.97	4.69 (2.50)	4.66 (2.58)	.96	1.29
Median (interquartile range)	4.76 (2.90-6.60)	4.79 (2.90-6.40)		4.15 (2.70-6.40)	4.35 (2.70-6.50)		
Total thoracic radiation dose, Gy							
Mean (SD)	50.4 (8.6)	49.5 (7.3)	.71	50.7 (8.8)	49.6 (7.5)	.62	13.57
Median (interquartile range)	45.0 (45.0-70.0)	45.0 (32.4-70.0)		45.0 (45.0-70.0)	45.0 (32.4-70.0)		
Response to primary treatment							
Complete	36 (51.4)	70 (71.4)	.03	35 (53.8)	27 (71.1)	.21	NA
Partial	28 (40.0)	23 (23.5)		24 (36.9)	8 (21.1)		
None or progression	6 (8.6)	5 (5.1)		6 (9.2)	3 (7.9)		

Abbreviations: NA, not applicable; PCI, prophylactic cranial irradiation.

^a^ The data set for the response to radiation therapy variable is incomplete because some follow-up information is unknown; thus, missing data were excluded for the calculation of these proportions.

After propensity score matching, the PCI and no-PCI cohorts were balanced, with 84 patients in each group, and were not statistically significantly different in terms of age (median [range] age, 65.0 [44.0-85.0] years vs 67.5 [40.0-86.0] years), sex (49 men [58.3%] vs 47 men [56.0%]), Eastern Cooperative Oncology Group performance status of 0 or 1 (71 patients [84.55] vs 72 patients [85.7%]), chemotherapeutic regimen (cisplatin or etoposide, 29 patients [82.9%] vs 32 patients [82.1%]), tumor size (mean [SD] diameter, 4.66 [2.50] cm vs 4.69 [2.58] cm), thoracic radiation dose (median, 45.0 Gy for both groups; range, 45.0-70.0 Gy for the PCI group and 32.4-70.0 Gy for the no-PCI group), smoking history (46 smokers [74.2%] vs 60 smokers [88.2%]), and response to radiation therapy (complete or partial response, 35 patients [79.1%] vs 59 patients [90.7%]) (Table 1). The standardized differences for all covariates were less than or equal to 13.57% in the postmatching cohort.

**Figure 1.**
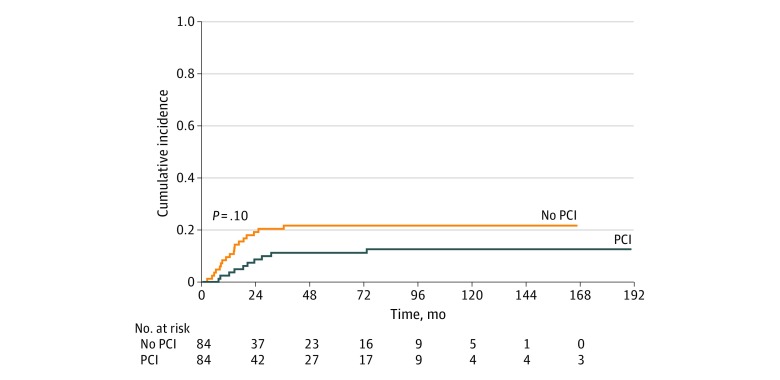
Cumulative Incidence Rate of Distant Brain Metastasis, With Death as Competing Risk, Among Patients With Limited-Stage Small Cell Lung Cancer Unadjusted analysis was performed on a propensity-matched sample of patients who did or did not undergo prophylactic cranial irradiation (PCI).

The 3-year unadjusted cumulative incidence rate of BM was higher in the no-PCI group than in the PCI group, when counting death as a competing risk, but the difference was not statistically significant (20.40% [95% CI, 12.45%-29.67%] vs 11.20% [95% CI, 5.40%-19.20%]; Gray test, *P* = .10) (Figure 1). A multivariable proportional Fine and Gray hazards model was also fitted to assess the association of PCI with BM, with the adjustment of tumor size and thoracic radiation dose in the model in the competing risk setting. The hazard ratio of 0.513 (95% CI, 0.239-1.098; *P* = .09) indicated that the use of PCI was not associated with a decrease in the risk of developing new BM.

**Figure 2.**
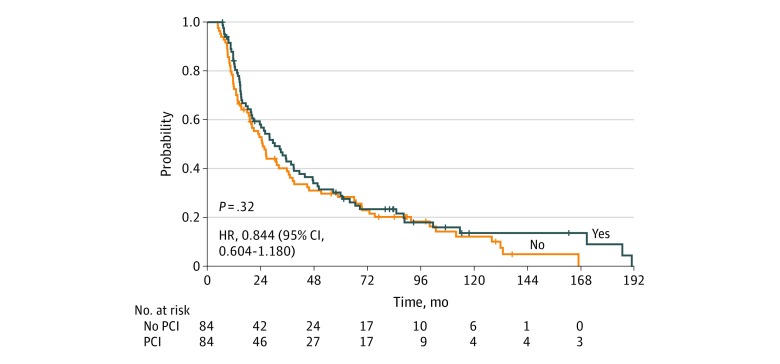
Comparison of Overall Survival in Propensity-Matched Patients With Limited-Stage Small Cell Lung Cancer Who Did and Did Not Undergo Prophylactic Cranial Irradiation (PCI) HR indicates hazard ratio.

An unadjusted survival analysis of the propensity-matched sample shows that OS was not statistically significantly different between the PCI and no-PCI groups after propensity score matching (HR, 0.844; 95% CI, 0.604-1.180; *P* = .32) (Figure 2). To verify, a multivariate Cox proportional hazards model was fitted on the data after propensity score matching to assess the association between PCI status and OS with the adjustment of other prognostic covariates in the model, including age (HR, 1.026; 95% CI, 1.005-1.046; *P* = .01), sex (HR, 1.421; 95% CI, 0.996-2.028; *P* = .05), Eastern Cooperative Oncology Group status 1 vs 0 (HR, 0.992; 95% CI, 0.635-1.550; *P* = .97), tumor size (HR, 1.010; 95% CI, 0.934-1.092; *P* = .81), and radiation dose (HR, 0.990; 95% CI, 0.968-1.012; *P* = .36). After adjustment, PCI was not associated with improved OS (HR, 0.787; 95% CI, 0.558-1.110; *P* = .17) (Table 2).

**Table 2.** Multivariable Cox Regression Model for Overall Survival, After Propensity Score Matching

Covariate	HR (95% CI)	*P* value
Age	1.026 (1.005-1.046)	.01
Sex, male vs female	1.421 (0.996-2.028)	.05
Eastern Cooperative Oncology Group performance status		
1 vs 0	0.992 (0.635-1.550)	.97
2 vs 0	1.837 (1.002-3.365)	.05
3 vs 0	0.307 (0.067-1.399)	.13
Tumor size	1.010 (0.934-1.092)	.81
Radiation dose	0.990 (0.968-1.012)	.36
Prophylactic cranial irradiation, yes vs no	0.787 (0.558-1.110)	.17

Abbreviation: HR, hazard ratio.

## Discussion

In this study, we found that for unselected patients with LS-SCLC undergoing contemporary head imaging, PCI was not associated with a decrease in the risk of developing BM or with improved OS. The 3-year incidence of BM was nearly doubled in patients who did not undergo PCI, at 20.40% vs 11.20%, compared with those who did undergo PCI, but the difference was not statistically significant.

Contemporary use of MRI and head computed tomography staging detects BM in approximately one-quarter of patients with SCLC at diagnosis and in an additional one-third of patients after initial therapy.^6^ Accordingly, in extensive-stage SCLC, it is now presumed that a meaningful proportion of patients enrolled in the European Organisation for Research and Treatment of Cancer trial had undetected BMs because head imaging was not required at baseline, which would have skewed the benefit-to-risk ratio of treatment in favor of PCI compared with the Japanese trial,^1^ which mandated central nervous system staging and surveillance. It could be reasonably argued that the same bias would apply to the LS-SCLC literature and the meta-analysis by Aupérin et al.^5^ Prior phase 3 trials^10^ that have shown reduced incidence of BM and also improvement in OS may not reflect the benefit of PCI in the modern era, which requires demonstration of no evidence of intracranial metastasis on the basis of brain MRI; these results will need to be confirmed in modern trials. For these reasons, new data suggest that practice patterns regarding the use of PCI in extensive-stage SCLC has been decreasing, with an increasing number of radiation oncologists recommending MRI surveillance as an alternative.^11^

Contemporary improvements in imaging and salvage techniques for BMs have also advanced, including stereotactic radiosurgery. Although the neurotoxicity associated with whole-brain radiation therapy and/or PCI is difficult to quantify, in some studies, PCI has been associated with substantial neurotoxicity compared with observation, as well as worse quality of life, particularly in older patients.^12^ It is because of this neurotoxicity that stereotactic radiosurgery is now the standard of care in favor of whole-brain radiation therapy for selected patients with a limited number of BMs, and randomized trials have been completed to use medications and/or conformal radiation techniques to avoid the hippocampus, both with the objective to minimize neurocognitive decline.^13,14^ However, the clinical decision is complex, and the resulting toxic effects associated with PCI must be weighed against the potential decline in the incidence of new BM, which can be associated with neurological symptoms and quality-of-life detriment.

Growing evidence from multiple retrospective studies^15,16,17^ suggests that MRI surveillance and stereotactic radiosurgery may be considered as a first-line option for selected patients with SCLC, and in the era of increasingly aggressive salvage stereotactic radiosurgery and new immunotherapeutic options for extensive-stage SCLC, this is now the focus of multiple prospective clinical trials. Synthesizing the available modern data would suggest that in regard to central nervous system radiation therapy recommendations, SCLC and non-SCLC should not be treated as distinctly as historically thought.

### Limitations

This study has limitations. Its design is retrospective, although we attempted to control for potential confounders through propensity score matching, and balance was well achieved. Furthermore, we did not collect data regarding the rates of salvage therapy in our no-PCI cohort. The use of PCI for patients with LS-SCLC is complex and is a personalized decision that should include discussion regarding the context of the survival benefit seen in the historical data, the consequence of the potential intracranial control benefit, patient understanding, the availability of various radiation salvage options, and the associated neurotoxic effects of whole-brain radiation therapy. Prospective studies are warranted.

## Conclusions

After using propensity score matching to minimize selection bias, we found that in patients with LS-SCLC undergoing contemporary head imaging, the use of PCI after thoracic radiation was not associated with a decrease in the risk of developing new BM. The use of PCI was not associated with an OS benefit.
